# Intermittency study of global solar radiation under a tropical climate: case study on Reunion Island

**DOI:** 10.1038/s41598-021-91639-9

**Published:** 2021-06-09

**Authors:** Qi Li, Miloud Bessafi, Peng Li

**Affiliations:** 1 Guangxi University of Science and Technology, 268 Avenue Donghuan, Chengzhong District, Liuzhou, Guangxi China; 2 ENERGY Lab, Université de La Réunion, rue René Cassin, 97715 St Denis, France

**Keywords:** Solar energy, Scientific data

## Abstract

The arbitrary order Hilbert spectral analyses are applied to study the intermittency and multifractality of Global Horizonal Irradiation (GHI) based on one available high sampling rate of 1-year GHI records located at Saint-Denis (Moufia) over Reunion Island. The scaling exponents $\xi \left( q \right)$ is estimated through the arbitrary order Hilbert spectral analyses, and three parameters: Hurst exponent (*H*), the fractal co-dimension (*C*_1_), and Lévy parameter ($\alpha$) are taken to study the multifractal process of the GHI in the sub-daily, daily fluctuations and also in seasonal variations. A power law behaviour with a spectral exponent *β* = 1.68 close to the Kolmogorov spectrum is detected through Fourier spectrum analysis, which indicates that the sub-daily fluctuations of GHI are nonstationary. The scaling exponent *ζ*(*q*) is then estimated by the arbitrary order Hilbert spectral analysis and the multifractal properties is detected. The log-stable model parameters $H,{ }C_{1}$ and $\alpha$ characterize the concavity of the scaling exponent *ζ*(*q*) for analysing the intermittency of GHI. The classification method is applied to the daily GHI for analysing the distribution of the daily intermittency process and five classes with GHI and *K*_*b*_ are obtained.

## Introduction

The climate system has been strongly affected by human’s activities in recent years. Green-House gases (GHG) that are now higher than at any point in history due to the anthropogenic emissions. Climate changes have resulted in the changes of nature and human systems all over the world in recent decades, and these effects from the climate change feedback also increase the vulnerability of natural and human systems, especially for small islands. Islands are heterogeneous in geomorphology, culture, ecosystems, populations, and hence also in their vulnerability to climate change. Vulnerabilities and adaptation needs are as diverse as the variety of islands between regions and even within nation states, often with little climate adaptation occurring in peripheral islands. GHG emissions from most small islands are negligible in relation to global emissions, yet small islands will most probably be highly impacted by climate change^1^. The combination of island geography and economic types informs the extent to which adaptation and mitigation actions might interact^2^. The geography and location of islands affect their sensitivity to hydro-meteorological and related hazards such as cyclones, floods, droughts, invasive alien species, vector-borne disease, and landslides. It is necessary and urgent to conduct mitigation an adaptation to the risks posed by global climate change in small islands.

The most severe impacts of climate change may still be avoided if efforts are made to transform current energy systems. There are multiple means for lowering GHG emissions from the energy system, while still providing desired energy services^3^. Renewable energy sources have a large potential to displace emissions of GHG from the combustion of fossil fuels and thereby to mitigate climate change. Climate changes, energy demands and increasing government attention are all driving the development of renewable energy. Renewable energy sources (solar, wind, biomass, tides, geothermal heat…) play a role on providing energy services in a sustainable manner and, in particular, in mitigating climate change^4^. Among of those different kinds of renewable energy resources, solar energy is regarded as the most promising renewable energy resources. Solar radiation incident at the Earth’s surface is the principal energy source for life on the planet, and largely determines the climatic conditions of our habitats. Solar energy could be used directly for heating and lighting homes and other buildings, for generating electricity, and for hot water heating, solar cooling, and a variety of commercial and industrial uses^5^. In recent decades, the solar energy is made use of a lot in the photovoltaic (PV) industry for electricity generation.

The cloud is the primary factor determining the amount of solar radiation received at a given point on the Earth’s surface^6^. Clouds modulate solar irradiance in all time and space scales. Low, thick clouds primarily reflect solar radiation and cool the surface of the Earth. High, thin clouds primarily transmit incoming solar radiation^7^. The variability and unpredictability of solar energy makes it belong to intermittent energy sources. In the complex dynamical systems, intermittency is the irregular alternation of phases of apparently periodic and chaotic dynamics or different forms of chaotic dynamics^8^. Different methods used to measure this phenomenon might give different definitions of intermittency. In the area of solar radiation, Tarroja et al.^9^ concern the concept of intermittency as the same meaning as fluctuation. They define the concept of the intermittency as the change in the magnitude of the total irradiation on a surface over a given time interval, which agrees with the concept of variability. On the contrary, Davis et al.^10^ imported the notion of intermittency from turbulence. They analysed radiation data artificially generated from cloud data. The amplitude of the fluctuations or intermittency of solar irradiation could drastically increase in a few seconds or minutes in one place within one hour whereas other places would experience low fluctuations. This can take place at different time during the day. This is mainly due to intermittent cloud covering triggered by orographic turbulence, lee waves, and prevailing trade winds. Intermittency study with the arbitrary-order Hilbert spectral analysis is mainly conducted in the turbulence field^11–13^. It is rare applied to the solar radiation signal. The multifractal intermittency properties are presented and analysed through the arbitrary-order Hilbert spectral analysis which is the combination of the Empirical Mode Decomposition (EMD) and Hilbert spectral analysis (HSA). Three multifractal parameters (*H*, *C*_1_,${ }\alpha$) are got to represent the intermittency properties of solar radiation.

Reunion Island is a French overseas territory which lies at 21° 06′ South and 55° 32′ East. Due to its location and tropical climate, annual sunshine is in the range of 1400–2500 h and can reach the value of 2900 h, for an altitude lower than 400 m. The monthly daily radiation reaches more than 6.5 kWh/m^2^ during the wet season in some parts of the coastal region (altitude < 300 m). Average temperatures oscillate from 25 to 32 °C for coastal regions and from 15 to 22 °C for regions located above an altitude of 1500 m in the interior of the island. Reunion is a mountainous island with very complex topography. It is famous for the two volcanos: the ‘Piton des Neiges’ (3070 m) and the ‘Piton de la Fournaise’ (2632 m). And there are three major central depressed areas: the Cirques of Mafate in the northwest, Salazie in the northeast and Cilaos in the southwest. Due to these complex terrain, the Reunion Island has its own typical synoptic characters^14^. Because of heterogeneous and rapidly changing cloudiness, tropical islands, such as Reunion Island in the South-West Indian Ocean, have significant solar resource that is highly variable from day-to-day (intermittency). Thus, this paper is to conduct the intermittency analysis of the global solar radiation (GHI) over Reunion Island, which could provide the properties of GHI for its prediction in the next step.

This paper is organized as follows. “Dataset and methodology” describes the global solar radiation measurements used in this study and presents the arbitrary-order Hilbert spectral analysis methods, the multifractal intermittency properties and classification method. “Results” is dedicated to the presentation and the discussion of the results, which are then briefly summarized in “Conclusion and discussion”.

## Dataset and methodology

### GHI measurements over Reunion Island

There is a relative dense world-monitoring network using relatively inexpensive but reliable pyrheliometer and pyranometer instruments for broadband solar radiation measurements from the surface^15,16^. A pyranometer is a radiometer used to measure both the solar global horizontal irradiance (GHI) and diffuse horizontal irradiance (DHI) arriving at a particular location on the Earth’s surface. For the intermittency analysis in this paper, the data used is from the lab LE^2^P in the University of Reunion. Since 2010, LE^2^P has established the RCI_GS solar radiation network to provide GHI and DHI at 1-min intervals using SPN1, 2-in-1 pyranometer from Delta-T devices. The RCI_GS network consists of 12 ground-based stations (with a reference unit dating back to 2008). Each station includes meteorological capabilities in the form of a Vaisala WXT520 suite.

In this paper, the GHI data sampling per second at one SPN1 station over Reunion Island is used as study dataset. This SPN1 station is located at Saint Denis (Moufia), which provides GHI second data from 01-06-2016 to 31-05-2017. In order to highlight the fully developed intermittency during intraday, only [7:00–17:00] (local time, GMT + 4) time range has been selected. This corresponds to a time range which exclude sunrise and sunset time through the year and to avoid the measurement affected by natural mask especially at the beginning and ending of the day.

### Arbitrary order Hilbert spectral analysis method

The objective of this paper is to study the intermittency of GHI over Reunion Island and the multifractal properties of GHI time series. A new method called Arbitrary Order Hilbert Spectral Analysis^17,18^ is applied to the GHI measurements for this goal. The arbitrary-order Hilbert spectral analysis was proposed by Huang et al.^18^ to characterize the scale invariant property of solar radiation signals.

This method is the extension of Hilbert-Huang Transform (HHT)^19,20^ for estimating the scaling exponent ***ξ***(*q*) which is the principal value of the solar radiation fluctuations. The arbitrary-order Hilbert spectral analysis is the combination of the Empirical Mode Decomposition (EMD) and Hilbert spectral analysis (HSA). The EMD method provides an effective tool to decompose a signal into a collection of Intrinsic Mode Functions (IMF) that allow well-behaved Hilbert transforms for computation of physically meaningful time–frequency representation. The HSA is performed to each obtained IMF component extracted by the EMD method. The energy in a time–frequency space is estimated from the Hilbert spectrum, *H*(*ω*, *t*) = *A*^2^(*ω*, *t*).

#### Empirical mode deposition (EMD)

EMD is used to decompose the data into a sum of different time series (modes), each one having a characteristic frequency^19–23^. The modes are called Intrinsic Mode Functions (IMFs) which satisfy two conditions: (1) the difference between the number of local extrema and the number of zero-crossings must be zero or one; (2) the mean value of the envelope defined by the local maximum and the envelope defined by the local minimum is zero^19,20^ The EMD procedure is to decompose a signal into IMFs follows these algorithms^19,20^:

1. The local extremes of the signal *X*(*t*) are identified.
2. The local maxima are connected together forming an upper envelope *e*_*max*_(*t*), which is obtained by a cubic spline interpolation. The same is done for local minima, providing a lower envelope *e*_*min*_(*t*).
3. The mean is defined as *m*_1_(*t*) = (*e*_*max*_(*t*) + *e*_*min*_(*t*))/2.
4. The mean is subtracted from the signal, providing the local detail *h*_1_(*t*) = *X*(*t*) − *m*_1_(*t*).
5. The component *h*_1_(*t*) is then examined to check if it satisfies the conditions to be an IMF. If yes, it is considered as the first IMF and denoted *C*_1_(*t*) = *h*_1_(*t*). It is subtracted from the original signal and the first residual^19,20^, *r*_1_(*t*) = *X*(*t*) − *C*_1_(*t*) is taken as the new series in step (1). On the other hand, if *h*_1_(*t*) is not an IMF, a procedure called “shifting process” is applied as many times as needed to obtain an IMF. The shifting process is: *h*_1_(*t*) is considered as the new data; the local extrema are estimated, lower and upper envelopes are formed and their mean is denoted *m*_11_(*t*). This mean is subtracted from *h*_1_(*t*), providing *h*_11_(*t*) = *h*_1_(*t*) − *m*_11_(*t*). Then it is checked again if *h*_11_(*t*) is an IMF. If not, the sifting process is repeated k times, until the component h_1k_(t) satisfies the IMF conditions. Then the first IMF is *C*_1_(*t*) = *h*_1*k*_(*t*) and the residual *r*_1_(*t*) = *X*(*t*) − *C*_1_(*t*) is taken as the new series in step (1).

The above procedure is repeated for n times and then *n*IMFs are obtained, when *r*_1_(*t*) becomes monotonic function, no further IMF can be extracted. Thus the original signal *X*(*t*) is written as a sum of IMF modes *C*_*i*_(*t*) and a residual *r*_*n*_(*t*)^19,20^.

1 $${\text{X}}\left( {\text{t}} \right) = \mathop \sum \limits_{i = 1}^{N} C_{i} \left( t \right) + r_{n} \left( t \right).$$

The shifting process above should be stopped by a criterion^24,25^. This criterion has been accomplished by limiting size of the standard deviation (SD) between 0.2 and 0.3.

2 $$SD = \mathop \sum \limits_{0}^{r} \left[ {\frac{{\left| {d_{i - 1} \left( t \right) + d_{i} \left( t \right)} \right|^{2} }}{{d^{2}_{i - 1} \left( t \right)}}} \right].$$

#### Hilbert spectral analysis (HSA)

HSA is known to be a Hilbert transform applied to each IMF component extracted from the original signal by the EMD method. Let's *C*_*i*_(*t*) to be the IMF mode, HSA acts as a time frequency analysis for extracting the energy–time–frequency information from the data^19,20,26,27^. The Hilbert transform applied to one mode *C*_i_(*t*) is written as:

3 $$\tilde{C} = \frac{1}{\pi }{\text{P}}\mathop \smallint \limits_{0}^{\infty } \frac{{C_{i} \left( {t^{\prime}} \right)}}{t - t^{\prime}}dt^{\prime},$$

where *P* means the Cauchy principle value^26,27^.

Then the analytic signal *z* can be defined as:

4 $$z = C + j\tilde{C} = A\left( t \right)e^{j\theta \left( t \right)} ,$$

where C, $\tilde{C}$ is real and imaginary part of a signal respectively. Where

5 $$Am = \left| z \right| = \sqrt[2]{{C + \tilde{C}^{2} }}$$

and *θ*(*t*) = *arg*(*z*) = *arctan*$\tilde{C}$. *A*(*t*) is an amplitude time series and *θ*(*t*) is the phase of the mode oscillation^26^. Hence, the instantaneous frequency *ω* is determined from the phase:

6 $$\omega = d\theta /dt$$

within such approach and neglecting the residual, the original time series is rewritten as

7 $$X\left( t \right) = Re\mathop \sum \limits_{i = 1}^{N} A_{i} \left( t \right)e^{{j\theta_{i} \left( t \right)}} ,$$

where *A*_*i*_ and *θ*_*i*_ are the amplitude and phase time series of mode *i* and Re means real part^19,20^. For each mode, the Hilbert spectrum is defined as the square amplitude *H*(*ω*,*t*) = *A*^2^(*ω*,*t*). *H*(*ω*,*t*) gives a local representation of energy in the time frequency domain. Then the Hilbert marginal spectrum *h*(*ω*) is written as:

8 $$h\left( \omega \right) = \mathop \smallint \limits_{0}^{\infty } H\left( {\omega ,t} \right)dt.$$

This analysis is similar to the Fourier spectrum, as it corresponds to the energy associated to the frequency. The generalized Hilbert marginal spectrum for the arbitrary-order statistical moment *q* ≥ *0* is defined as^19,20^:

9 $$L_{q} \left( \omega \right) = \mathop \smallint \limits_{0}^{\infty } p\left( {\omega ,t} \right)A^{q} dA.$$

Hence the scale invariance in the Hilbert space is written as:

10 $$L_{q} \left( \omega \right)\sim \omega^{ - \xi \left( q \right)} ,$$

where ${ }\xi \left( q \right)$ is the corresponding scaling exponent in the Hilbert space. This scaling exponent function is linked to scaling exponent function *ζ*(*q*) of structure functions analysis by the expression^17^:

11 $$\zeta \left( q \right) = \xi \left( q \right) - 1.$$

### The multifractal intermittency properties

In this paper, we focus on the log-stable model or universal multifractal proposed by Schertzer and Lovejoy^28^, and Kida^29^:

12 $${\upzeta }\left( {\text{q}} \right) = qH - \frac{{C_{1} }}{{\left( {\alpha - 1} \right)}}\left( {q^{\alpha } - q} \right),$$

where *H* = $\zeta$(1) the Hurst parameter defines the degree of smoothness or roughness of the field. The parameter *C*_1_ is the fractal co-dimension of the set giving the dominant contribution to the mean (*q* = 1) and bounded between 0 and *d* (*d* the dimension space, here *d* = 1). It measures the inhomogeneity mean or the mean intermittency characterizing the sparseness of the field: the larger *C*_1_, the more inhomogeneous the mean field. The multifractal Lévy parameter *α* is bounded between 0 and 2, where *α* = 0 corresponds to the monofractal case and *α* = 2 corresponds to the multifractal lognormal case. The parameter *α* measures the degree of multifractality, i.e., how fast the inhomogeneity increases with the order of the moments^30,31^.

The computation of multifractal indices *α*, and *C*_1_ are obtained from equation before^32^:

13 $$R\left( q \right) = q\zeta^{\prime}\left( 0 \right) - \zeta \left( q \right) = \frac{{C_{1} }}{{\left( {\alpha - 1} \right)}}q^{\alpha } .$$

Thus, the function *R*(*q*) versus *q* will have a slope *α* and *C*_1_ can be estimated by the intercept.

Another way to characterize a multifractal process is the singularity spectrum *M*(*γ*). The singularity spectrum is a function used in multifractal analysis to describe the fractal dimension of a subset of points of a function belonging to a group of points that have the same Hurst exponent. Intuitively, the singularity spectrum gives a value for how fractal a set of points are in a function. Related to the scaling exponents $\zeta \left( q \right)$ presented above, *M*(*γ*) could be present:

14 $$\gamma = \frac{d\zeta \left( q \right)}{{dq}},$$

15 $$M\left( \gamma \right) = \gamma q - \zeta \left( q \right) + 1.$$

If it is a monofractal process, *γ* = *H* and *M*(*γ*) = 1.

### Daily solar radiation clustering

In order to analyze the distribution of the daily intermittency process, the classification method is applied to the daily GHI. Different class presents their intermittency characterization. There are many studies on classification methods conducted on solar radiation data. Muselli et al.^33^ applied a classification methodology with parameters defined from hourly clearness index profiles. Maafi and Harrouni^34^ and Harrouni et al.^35^ used fractal dimension and daily clearness index *kt* as classification parameters. Three classes obtained by some specific thresholds of these parameters correspond to clear sky, partially clouded and overcast sky, respectively. Soubdhan et al.^36^ classified daily distributions of the clearness index *kt* by estimating a finite mixture of Dirichlet distributions. This method didn’t assume any parametric hypothesis on the daily distributions in order to qualify the fluctuating nature of solar radiation under tropical climate. Bessafi et al.^37^ show interest and disadvantages of two approaches for classifying curves. The first is based on a vector representation of curves, the second offers the D'Urso and Vichi distance incorporating the mathematical properties of curves and based on the first and second finite derivatives. These two approaches are applied to the classification of sources of solar radiation. In this study, the method of Bessafi et al.^37^ is chosen to do clustering GHI and analyze the intermittency in different classes. This classification would be a first step to check the variability of intermittency for each type of weather and thus the range value of (*H*, *C*_*1*,_${ }\alpha$).

Bessafi et al.^37^ use the direct fraction noted as *K*_*b*_ to obtain the different classes:

16 $$K_{b} = 1 - K_{d} = 1 - \frac{{f_{diff} }}{{f_{glo} }} = \frac{{f_{dir} }}{{f_{glo} }},$$

where $f_{diff}$ means the diffuse part of the global solar radiation, and $f_{glo}$ means the global solar radiation. Thus, when direct faction note *K*_*b*_ is closed to 1, it means it is clear sky day and the surface could receive more solar energy. Otherwise, the *K*_*b*_ is closed to 0, which means it is cloudy day. And three data mining methodologies are applied in the classification: PCA, Ward and K-means clustering methods.

The dendrogram (clustering tree) of hierarchical ascending classification is performed on GHI records. The ascending hierarchical clustering creates a nested sequence of partitions of the patterns from a dissimilarity matric, and proceeds by series of fusions of the n objects into groups^38^. It produces a series of partitions of the data, *Pn*, *Pn *− 1… *P*1. At each stage, the ascending hierarchical clustering regroups the two clusters that are closest according to a Euclidean distance metrics.

## Results

### Mean sub-daily and daily multifractal pattern

**Figure 1:**
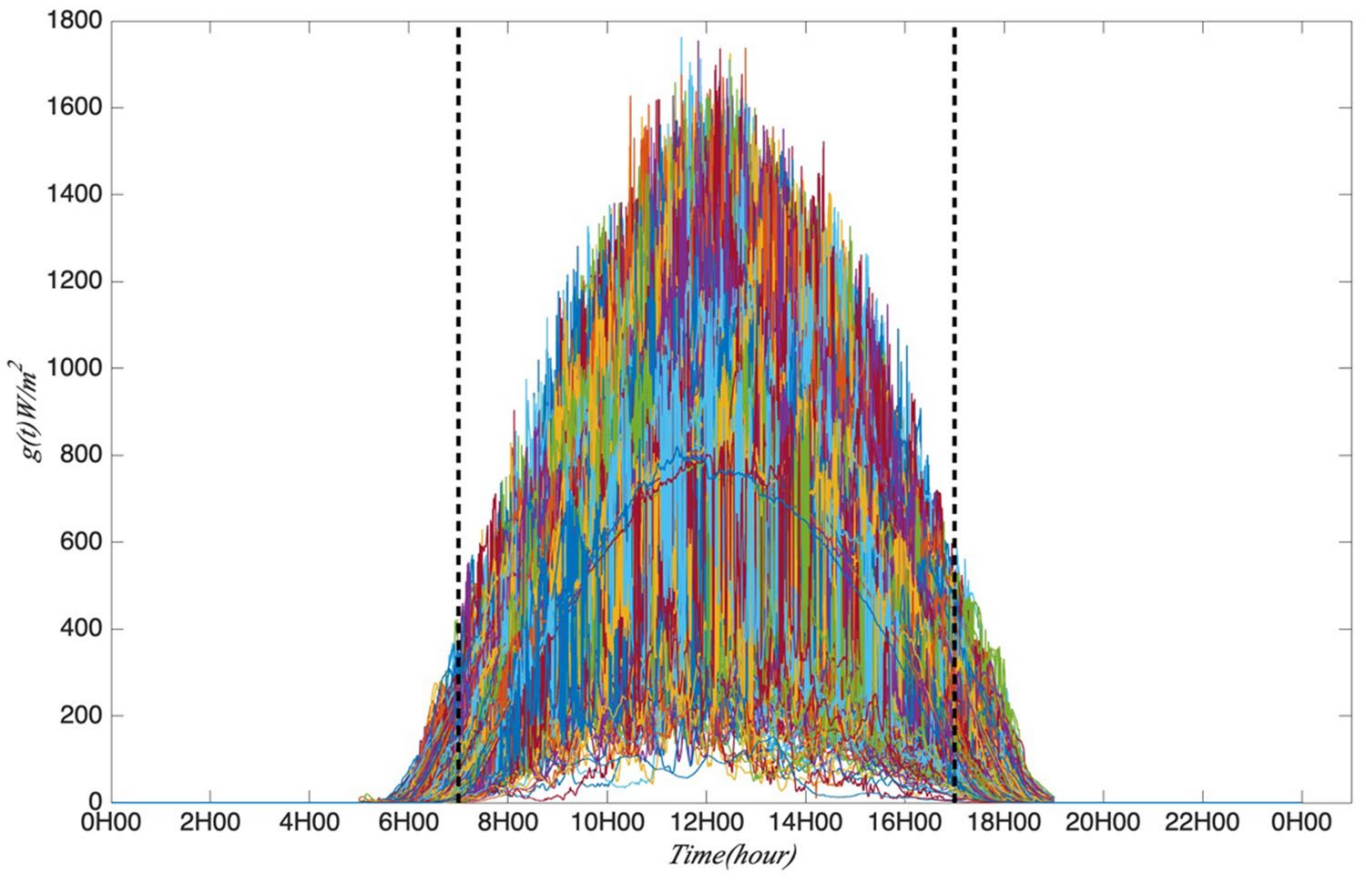
The daily global solar radiation GHI from 01-06-2016 to 31-05-2017. 7–17 h is the time period considered in this part.

**Figure 2:**
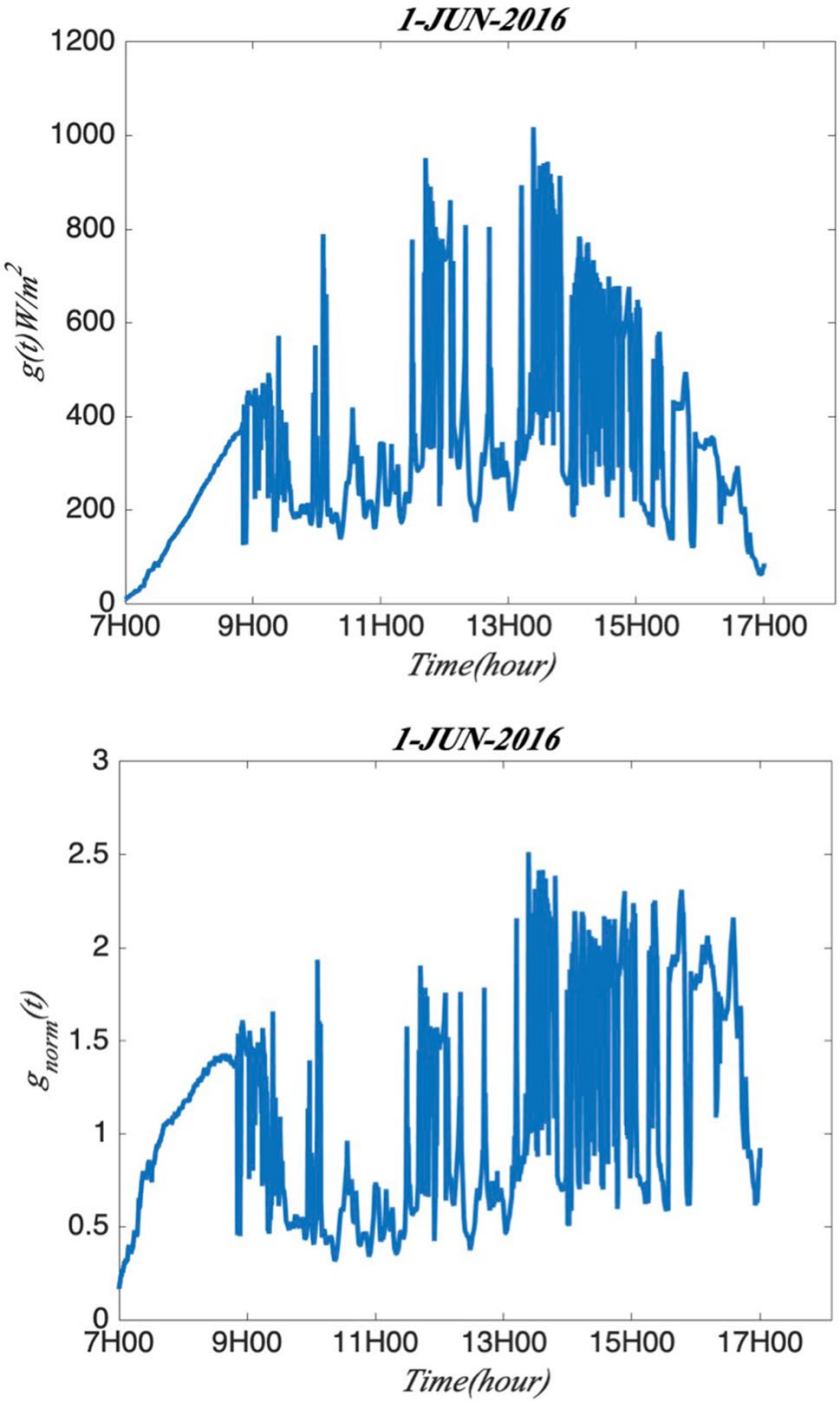
An example of typical daily global solar radiation sequence *g*(*t*) (panel up) and the corresponding normalized solar radiation sequence *gnorm*(*t*) (panel down) from 7 to 17 h on 1-June-2016.

As there is one SPN1 station at Moufia which records the GHI per second from 01-06-2016 to 31-05-2017, it would be interesting to study the intermittency and multifractality of GHI sampling at this high frequency. Firstly, the mean sub-daily and daily multifractal pattern of GHI is analysed. It is normal that the GHI during the day time (7–17 h) is much higher than at night. So, it is more interesting to focus on the GHI variability within the day time for checking the annual and seasonal cycle. The arbitrary order Hilbert spectral analysis (EMD + HSA) is applied to GHI time series for obtaining the generalized scaling exponent and log-stable model parameters are analysed. Then the multifractal processes could be found in the sub-daily and daily fluctuations. Figure 1 gives the distribution of the studied dataset, which shows the daily variability of GHI for all the days. Figure 2 presents one day’s global solar radiation from 7 to 17 h on 1 June 2016 and the corresponding normalized time sequence as an example. These two plots show the short time scales of this daily global solar radiation and exhibit the fluctuations stochastically distributed in time (seconds). The normalized time sequence is used as input data to the following intermittency and multifractality analysis. This part gives mean sub-daily and daily multifractal pattern of global solar radiation at one station with second dataset.

#### Fourier spectrum analysis: power law and Kolmogorov spectrum

In the field of solar energy, the variability of a stochastic process as GHI is often characterized by a second order statistic such as the Fourier spectrum *E*(*f*)^39^. The power spectral density separates and measures the amount of variability occurring in different frequency bands. Scale invariance can be detected by computing *E*(*f*). The objective of using Fourier spectrum analysis here, is to detect if there is a law scale in the GHI time series. For a scale invariant process, the following power law is obtained over a range of frequencies *f*:

17 $${\text{E}}\left( f \right)\sim f^{ - \beta } ,$$

where *β* is the spectral exponent which contains information about the degree of stationarity or non-stationarity inside a time series^40,41^:

- *β* < 1, the process is stationary;
- *β* > 1, the process is nonstationary;
- 1 < *β* < 3, the process is nonstationary with increments stationary.

**Figure 3:**
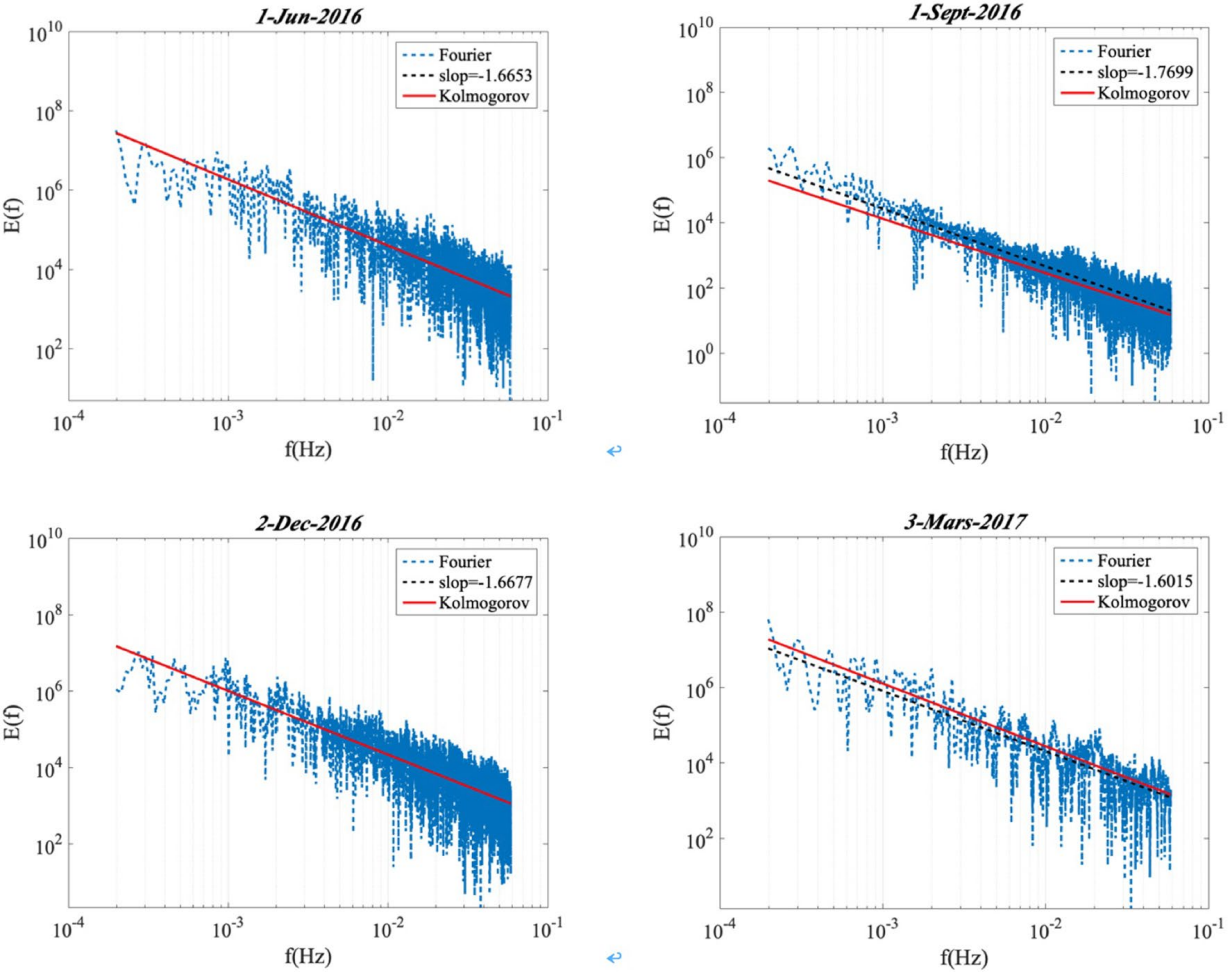
The power density spectrum *E*(*f*) of the normalized time sequence on 4 different days (01-June-2016, 01-September-2016, 02-December-2016, and 03-Mars-2017) displaying a power law behavior (the dash black line). Fourier spectrum also displaying a power law behavior close to the Kolmogorov spectrum (red line).

In Figure 3, the Fourier spectrum *E*(*f*) of the normalized global solar radiation *gnorm*(*t*) is given in log–log scale for four different days (01-June-2016, 01-September-2016, 02-December-2016 and 03-Mars-2017). These four days present the winter season (June), winter-to-summer transfer season (September), the summer season (December), and the summer-to-winter transfer season (Mars). The spectrums for these four days all display a power law behaviour (the dash black line) close to the Kolmogorov spectrum (*β* = − 5/3) for frequencies 1.8 × 10^–4^ ≤ f ≤ 0.07 Hz, corresponding to time scales 14 ≤ T ≤ 5555 s (approximately 1.54 h). Fourier spectrum also displays a power law behavior close to the Kolmogorov spectrum (red line).

**Figure 4:**
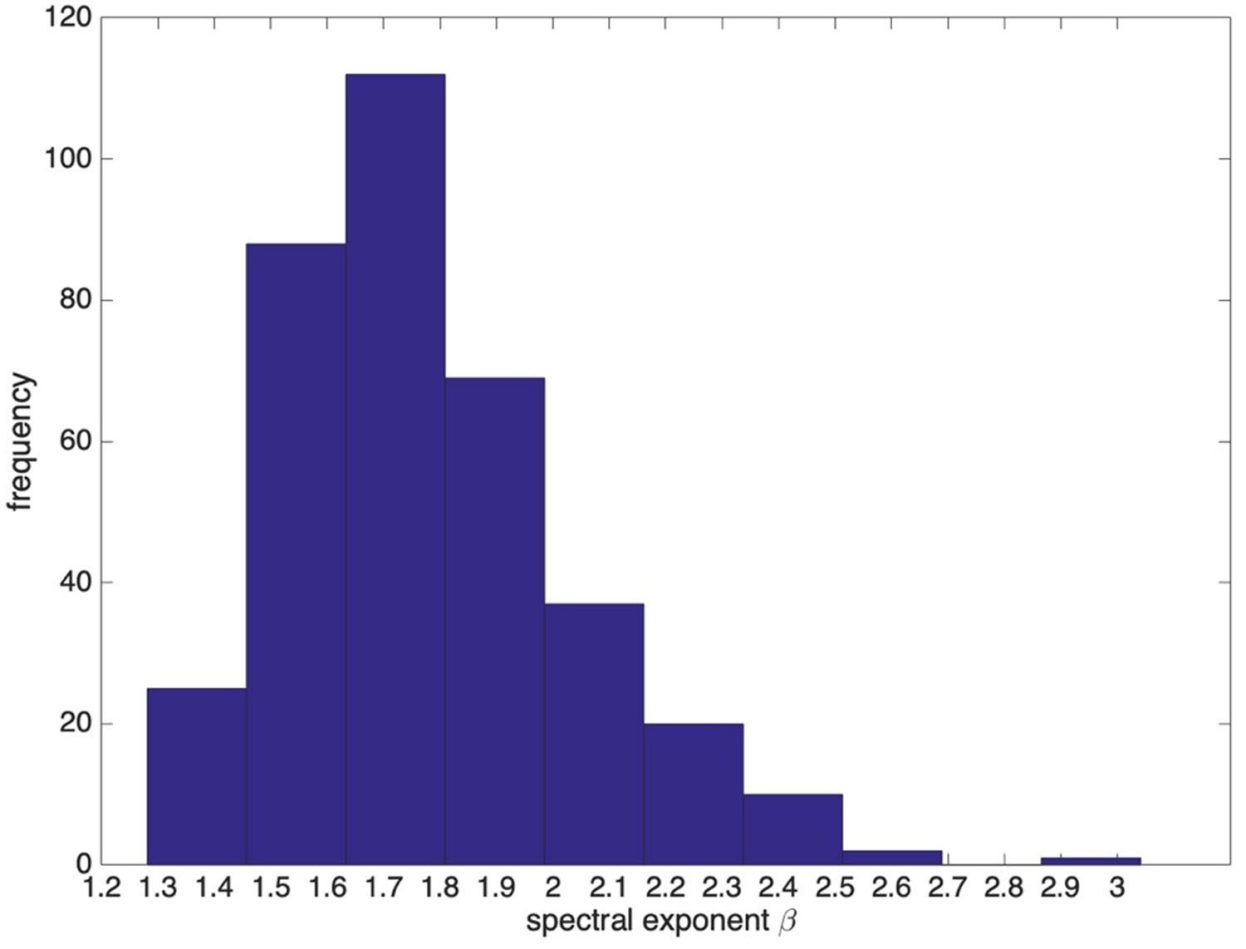
Histogram of the spectral exponent *β* estimated from Fourier spectra from June 2016 to May 2017.

**Figure 5:**
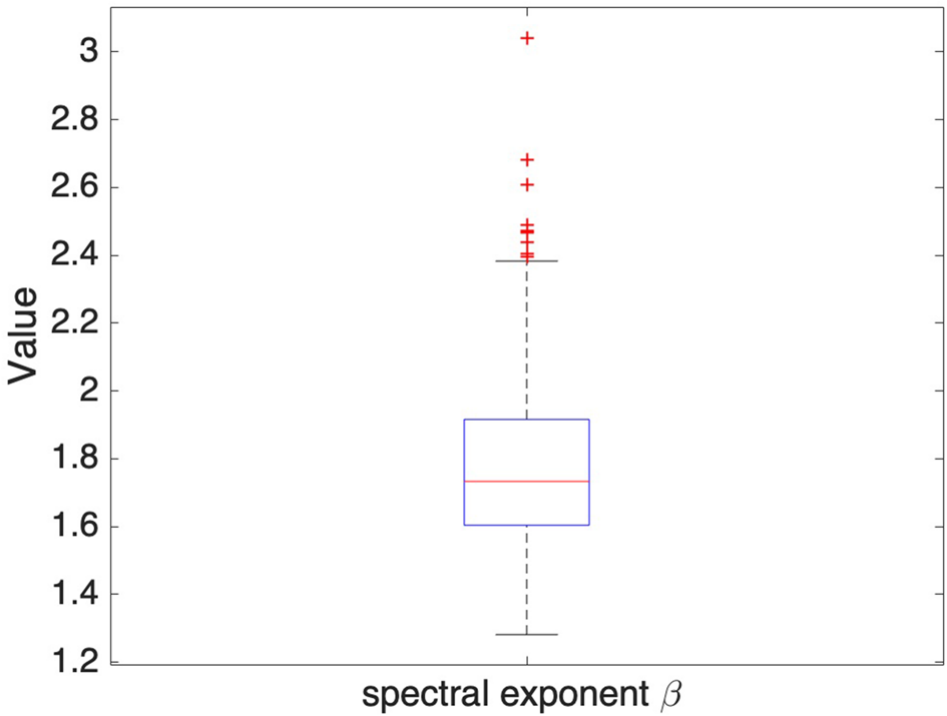
The boxplot of the spectral exponent *β* estimated from Fourier spectra from June 2016 to May 2017.

Figure 4 illustrates the histogram of the spectral exponent *β* estimated separately for the 1 year, June 2016 to May 2017. The idea is to emphasize that even if the spectral exponent is highly variable throughout the year as shown with 4 selected days of selected season, the distribution is bounded and centred around a mean value -1.68. The spectral exponent ranges between 1.3 and 2.7. The Fourier spectrum displays a power law behavior with 1.46 < *β* < 1.86 for 63% of the time sequences and *β* > 2 for 18% of sequences. It means that all the sub-daily fluctuations are the time nonstationary fluctuations (*β* > 1). Figure 5 presents the boxplot of the spectral exponent *β* estimated from Fourier spectra in from June 2016 to May 2017, which shows that the spectral exponent is centred around 1.7.

As the analysis was presented above, the spectral exponent *β* also presents a power law scale when comparing with other well-known nonstationary case as Kolmogorov shown for homogeneous turbulence (*β* = 5/3 ~ 1.66). However, it is still necessary to go further checking what kind of non-stationarity it is in these sub-daily fluctuations with multifractal analysis.

#### Generalized scaling exponent ζ$\left( q \right)$

**Figure 6:**
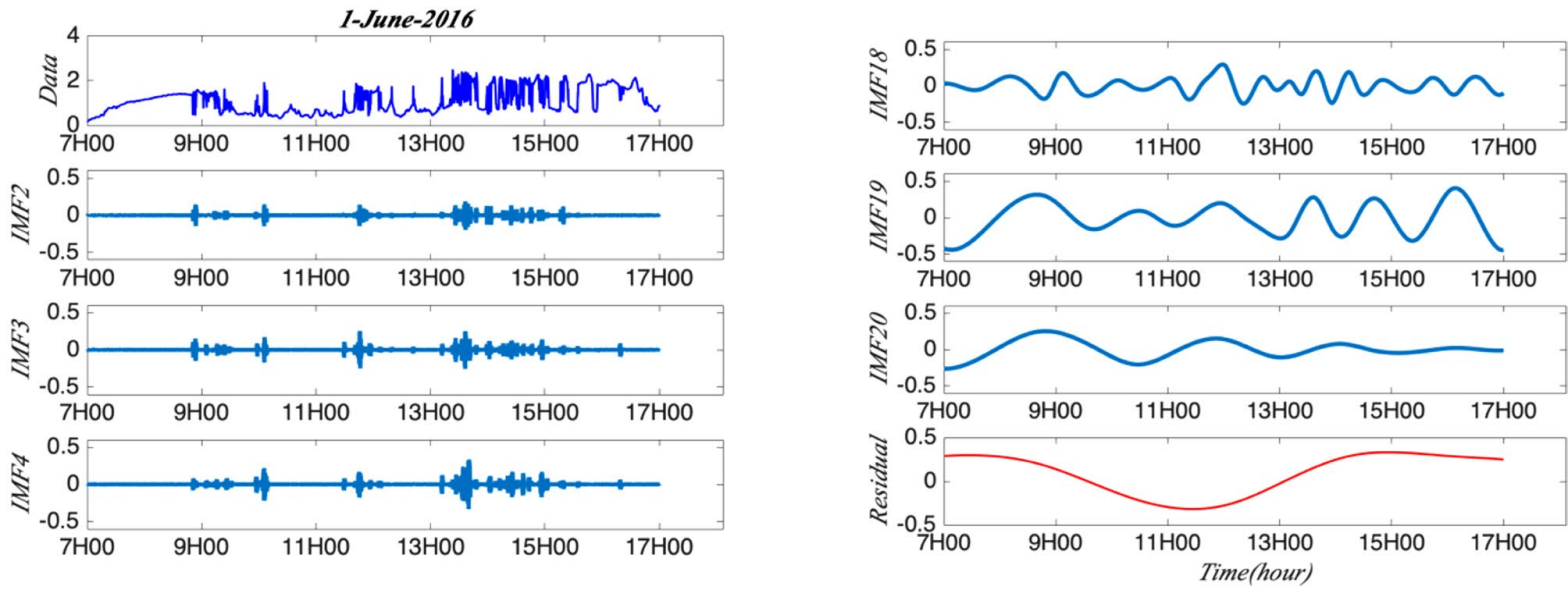
Decomposition of the signal *gnorm*(*t*) on 1-June-2016 into 20 IMF modes with one residual (IMF 4–17 is not list).

The arbitrary order Hilbert spectral analysis (EMD + HSA) is then applied to the normalized global solar radiation fluctuations *gnorm*(*t*) for estimating the scaling exponent ζ(q). The generalized exponent is obtained to see whether the sub-daily fluctuations are monofractal or multifractal processes. Figure 6 displays the EMD decomposition process into IMF modes and residual and it gives some idea on how intermittency triggers the GHI fluctuations.

**Figure 7:**
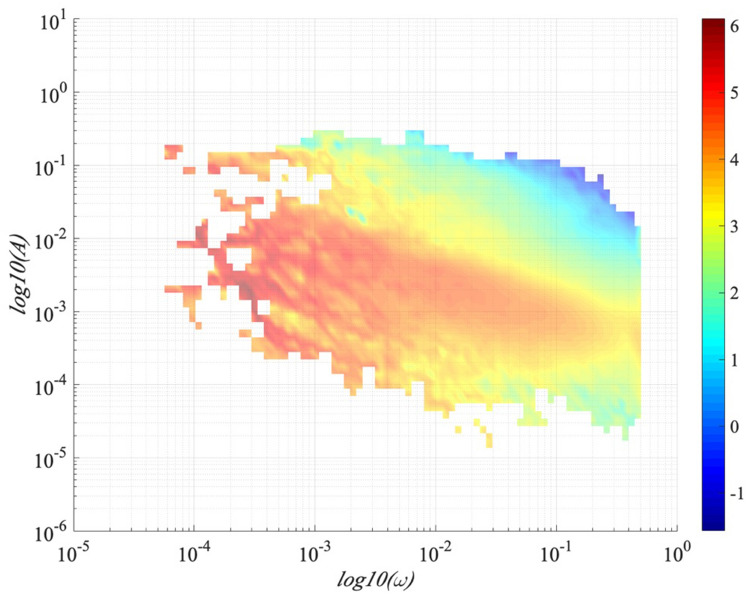
The probability density function, PDF *p* (*ω*,* A*) (in log scale) of the GHI recorded on 1-June-2016 in an amplitude-frequency space.

**Figure 8:**
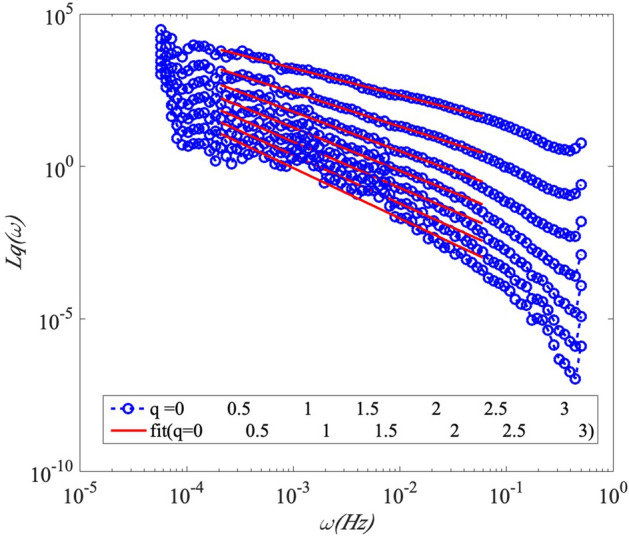
Arbitrary order Hilbert marginal spectrum *Lq*(*ω*) displaying a scaling in log–log plot in different orders of moments (*q* = 0, 0.5, 1, 1.5, 2, 2.5, 3). For 0 ≤ *q* ≤ 3 with 0.5 increment and for frequencies 1.8 × 10^–4^ ≤ *f* ≤ 0.07 Hz, corresponding to time scales 14 s ≤ *T* ≤ 5555 s on 1-June-2016. Power law behaviour is observed on the inertial range.

Figure 7 presents the probability density function (PDF), *p *(*ω*, *A*) of the GHI record on 1-June-2016, which is the first 2D amplitude-frequency representation of GHI fluctuations. The amplitudes decrease with increasing frequencies with a scaling trend. For checking if there is a scaling law in the inertial range, the Arbitrary order Hilbert marginal spectrum displays a scaling in log–log plot in Figure 8. The plots in different orders of moment (*q* = 0, 0.5, 1, 1.5, 2, 2.5, 3) are fitted by a least square indicating the scaling relationships. For frequencies 1.8 × 10^–4^ ≤ *f* ≤ 0.07 Hz (corresponding to time scales 14 s ≤ *T* ≤ 5555 s), *q* = 0, 0.5, 1, 1.5, 2, 2.5, 3 with 0.5 increment. The power law behaviour is observed on the inertial range in the marginal spectrum. Each arbitrary-order statistical moment *q* reveals a law scale in the inertial range with a scaling exponent $\xi \left( q \right)$ which corresponds to the slope of the straight line in the log–log plot.

**Figure 9:**
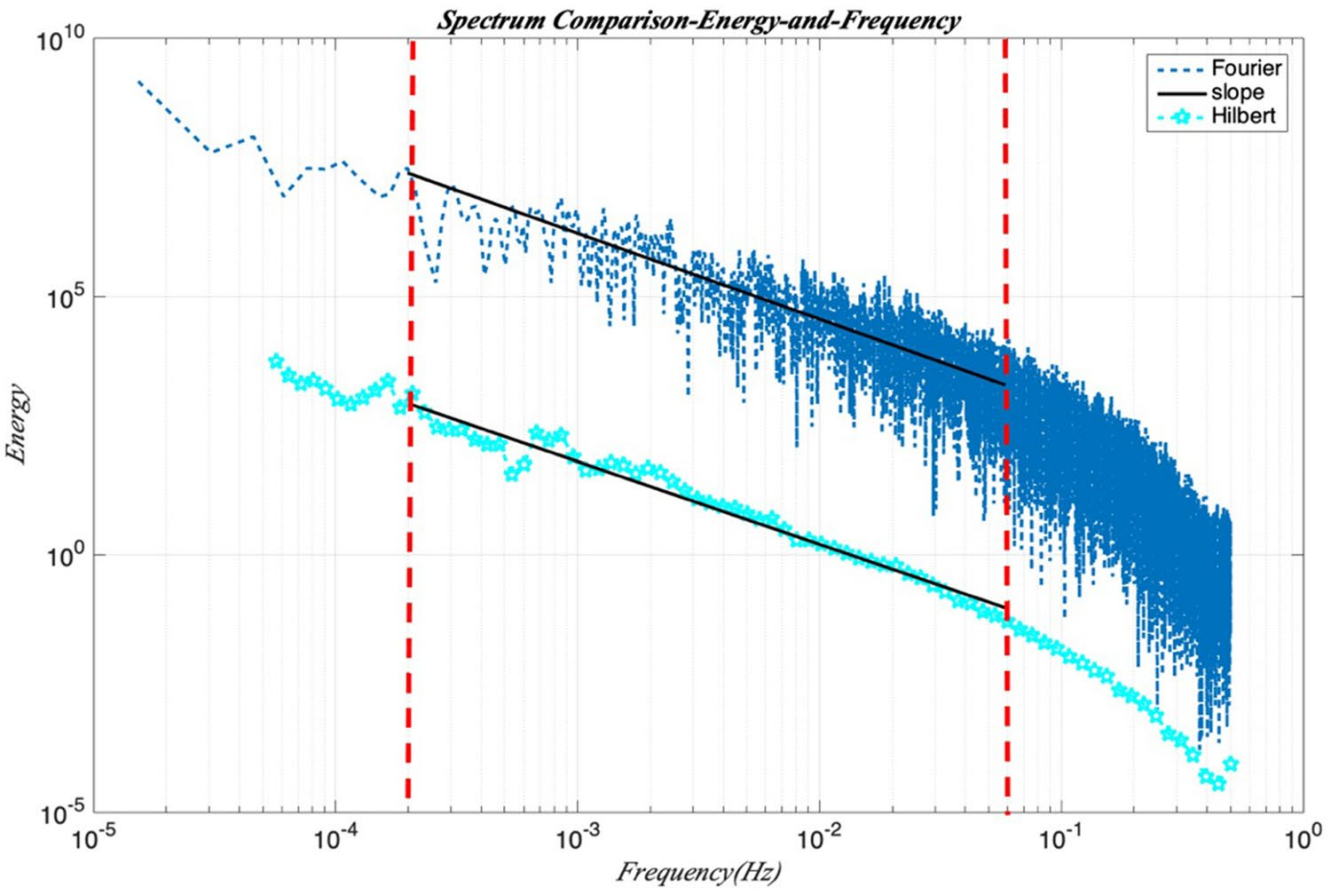
Comparison of the Hilbert marginal spectrum and Fourier spectrum for GHI at Station Moufia. A power law behavior is observed in the range of frequency 1.8 × 10^–4^ ≤ *f* ≤ 0.07 Hz, corresponding to time scales 14 ≤ *T* ≤ 5555 s: this range is marked by the vertical black lines.

The Hilbert marginal spectrum is also compared to Fourier spectrum in Figure 3 on 1-June-2016. Figure 9 shows that the Hilbert marginal spectrum can reproduce well the power law as Fourier spectrum which is the reference for spectral analysis. The Hilbert marginal spectrum could catch the same spectral feature as Fourier technique. Hilbert marginal spectrum could capture the power law behaviour (marked by the vertical black lines) on the range of frequency 1.8 × 10^–4^ ≤ *f* ≤ 0.07 Hz as Fourier spectrum. This demonstrates the ability of Hilbert marginal spectrum for catching the power law inside time series.

**Figure 10:**
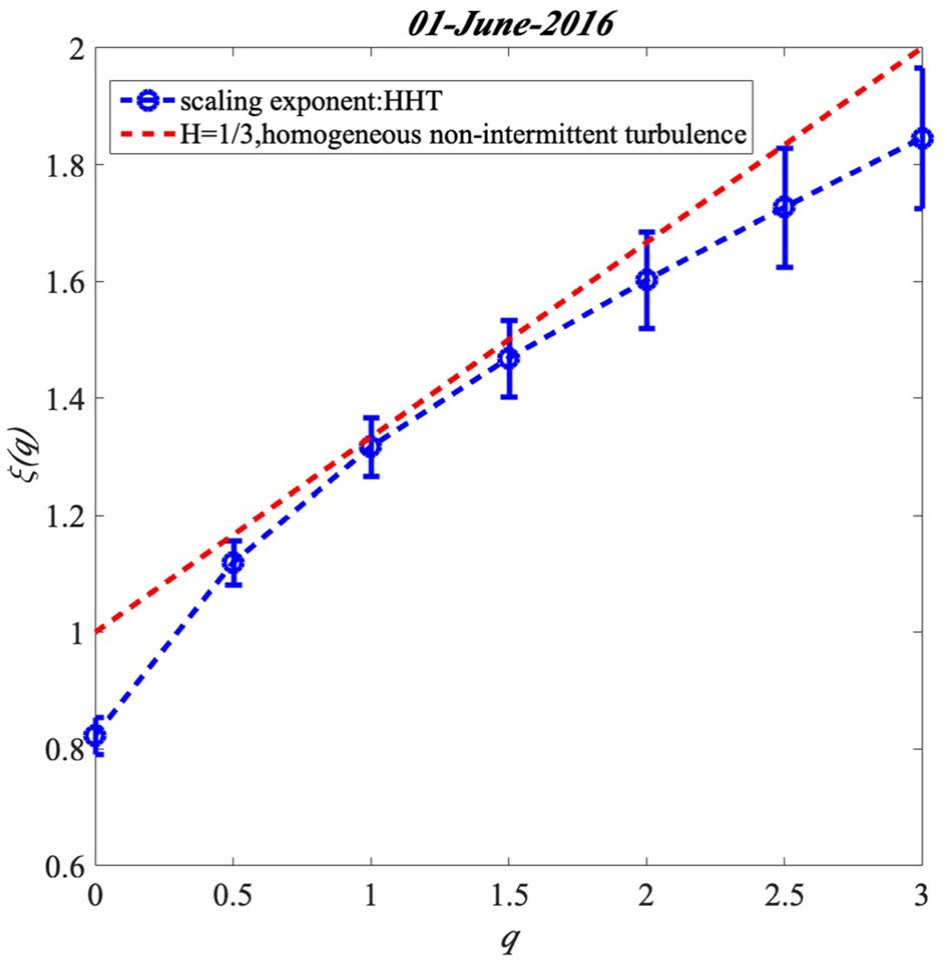
The scaling exponents calculated by the HHT compared to a non-intermittent linear model *qH* + 1 with H = 1/3 (dashed red line) the Hurst exponent.

The scaling exponents $\xi \left( q \right)$ of GHI at station Moufia on 01-June-2016 is shown as an example in Figure 10. This curve is calculated by the HHT and it is concave, which indicates the multifractal properties of the solar radiation. For comparison, a reference line *qH* + 1 with H = 1/3 (dashed red line) is shown in the figure, which corresponds to monofractal processes. The scaling exponents $\xi \left( q \right)$ in this figure is shown with error-bar plot, which uses 95% confidence of the error at each *q* for the scaling exponents ${ }\xi \left( q \right)$. This error-bar plot gives the mean of the scaling exponents ${ }\xi \left( q \right)$ in the middle point with the positive and negative variance in the up and down.

#### Log-stable model parameters $H, C_{1}$ and $\alpha$

**Figure 11:**
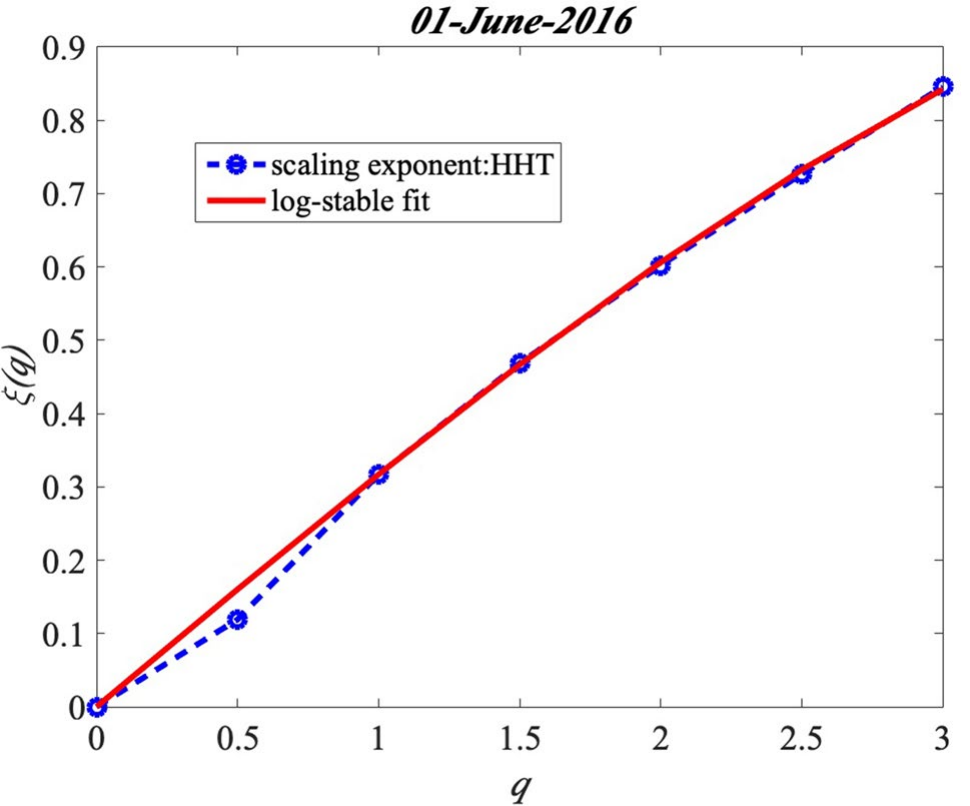
The scaling exponents (HHT) with log-stable fitting (red line) on 01-June-2016: *H* = 0.32, *C*_1_ = 0.03, *α* = 2.24.

**Figure 12:**
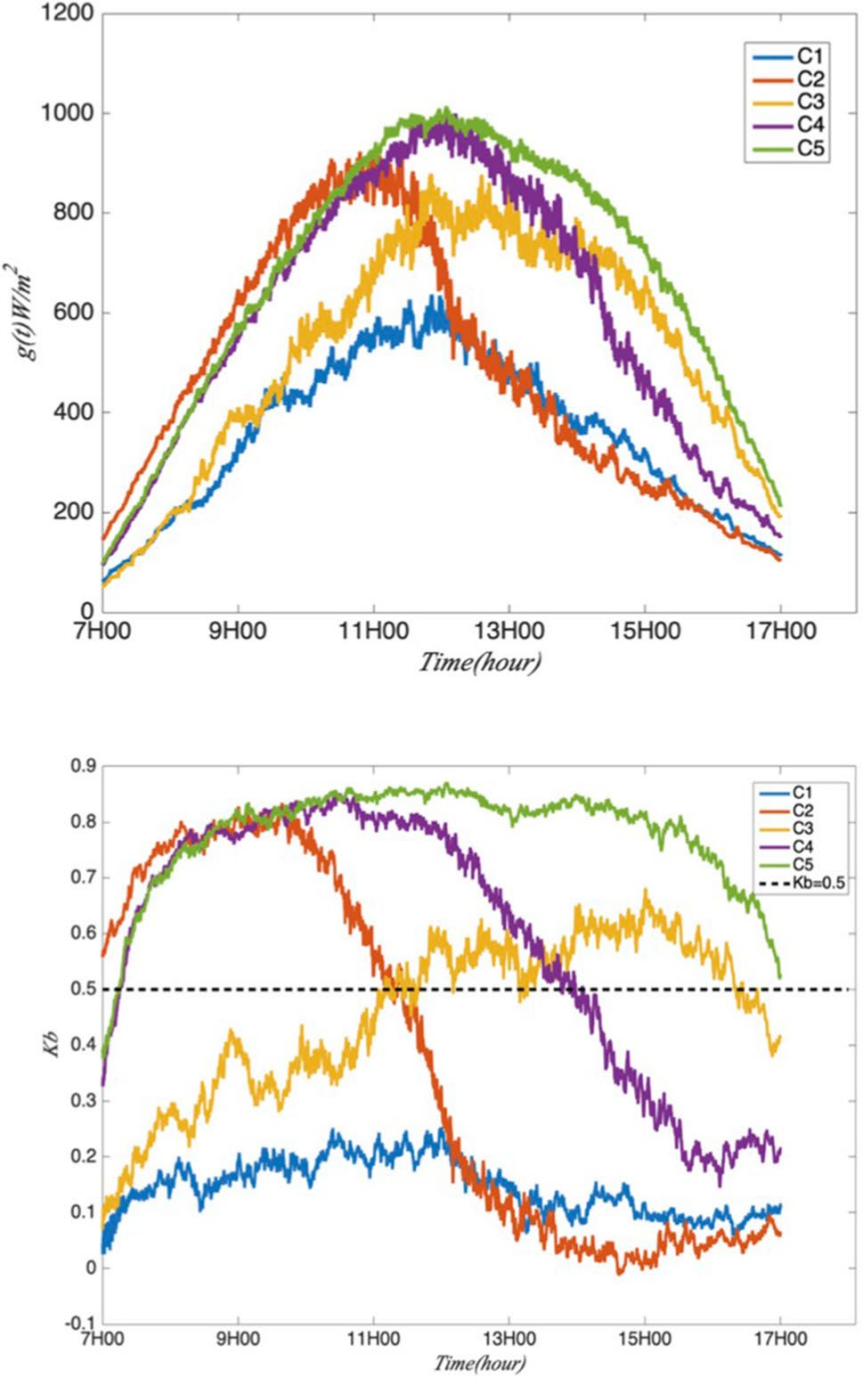
The classification for the GHI (panel up) and the *K*_*b*_ (panel down).

The generalized scaling exponent *ζ*(*q*) is obtained through the arbitrary order Hilbert spectral analysis in the last section. The multifractal process is detected for this sub-daily GHI fluctuation at station Moufia. Then this section is to characterize the concavity of the scaling exponent *ζ*(*q*) for analysing the intermittency of GHI. Figure 11 shows the log-stable fitting of GHI fluctuations on 01-June-2016. *H* = 0.32, *C*_1_ = 0.03, and *α* = 2.24 are estimated through the log-stable model. All these analyses are based on one day for sub-daily GHI multifractal pattern. Then, the study is extended to all days of the year June 2016 to May 2017 record for the daily GHI multifractal analysis, showing that the GHI fluctuations are highly variable from day-to day and thus the set of triplets (*H*, *C*_1,_${ }\alpha$) may be sensitive to this variability. In order to analyse the distribution of the daily intermittency process, the classification method is applied to the daily GHI. Different class could clearly present their intermittency characterization. Five classes with GHI and *K*_*b*_ are obtained in Figure 12.

Class 1 corresponds to a very low level of sunshine in all of the day. This class presents dominant local phenomena which include, on one hand, the weak trade winds accompanied by a flow of moisture leading to significant effects of orographic clouds, on the other hand, the land breeze phenomenon induced by thermal contrasts^42^. Class 2 has a sunny beginning until mid-morning around 09:00–09:30 and a cloudy afternoon. Diffuse radiation is dominant in the afternoon while the direct component is more important in the morning. Class 3 corresponds to the days with a huge variability with the variable weather. The performance of Class 4 is similar to that of Class 2, but with a stronger sunny regime during all morning until early afternoon. DHI predominates later in Class 4 than in Class 2. Class 5 corresponds to a regime of good weather throughout the day. Intermittent clouds passing over the station do not have a systematic character since direct radiation dominates in this class.

The multifractal parameters (*H*, *C*_1,_${ }\alpha$) then could be classified into these five classes as in Table 1. The mean of the multifractal parameters (*H*, *C*_1,_${ }\alpha$) in five classes are presented in the table. The mean value of *H* for class 1 (0.52) is larger than other classes and it is closer to 0.5, indicating that GHI fluctuations in five classes are anti-persistent in average and the class 1 is closer to random process. All the *C*_1_ mean values are smaller than 0.1 for the five classes and the class 1 and 2 have larger *C*_1_ than other classes indicating that the days in class 1 and 2 are more intermittent. $\alpha$ ∈ [1, 2] for class 1, 2 and 5 indicate that those days are multifractal with Levy generators and unbounded singularities.

**Table 1:** The mean of the multifractal parameters (*H*, *C*_1,_${ }\alpha$) in five classes.

	Class 1	Class 2	Class 3	Class 4	Class 5
*H*	0.52	0.40	0.37	0.38	0.38
*C*_*1*_	0.10	0.09	0.04	0.06	0.08
${ }\alpha$	1.63	1.56	2.37	2.06	1.88

**Figure 13:**
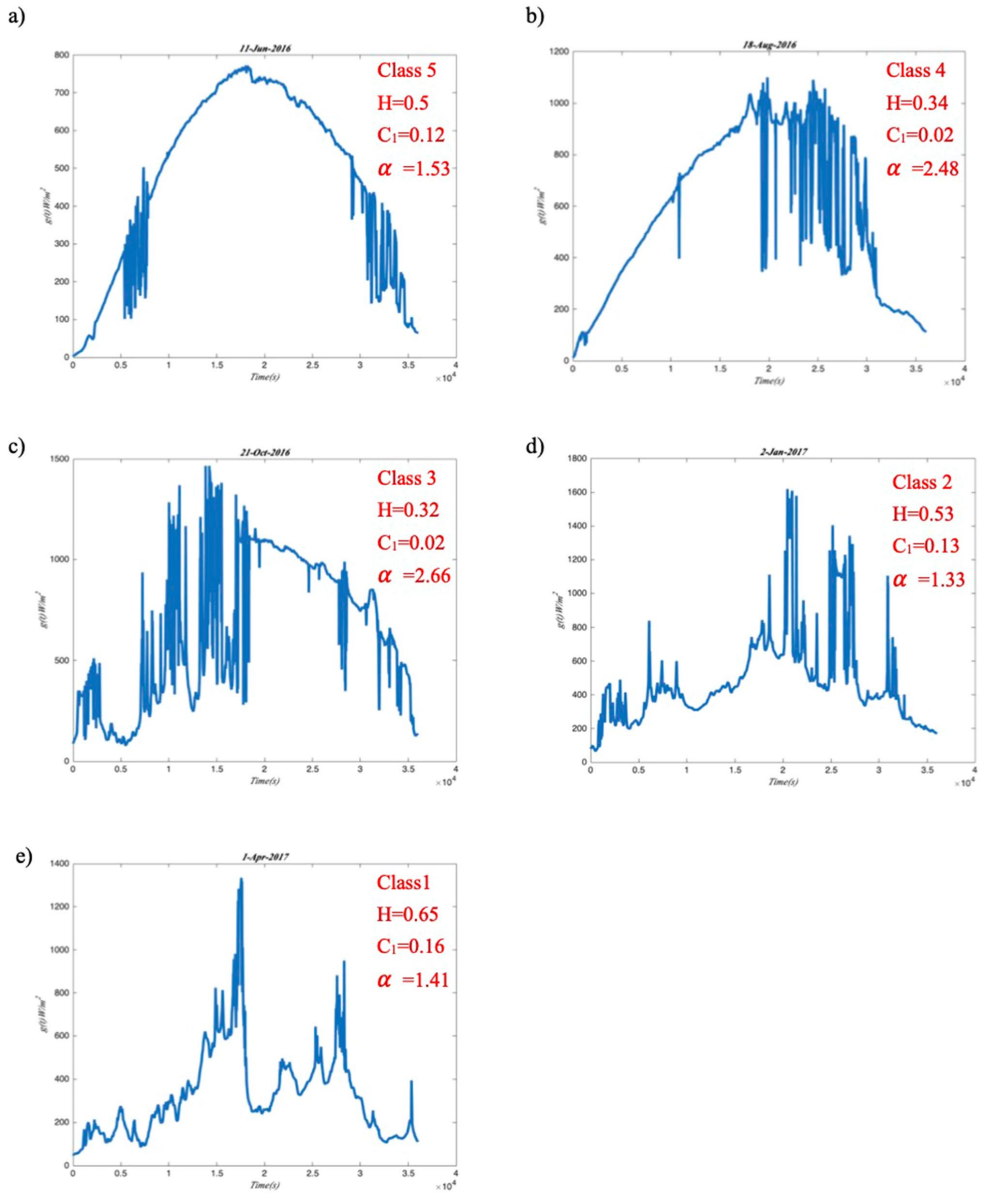
A few days of daily GHI sequences illustrating the varying effect of multifractal parameters, *H*, *C*_1_, and *α*.

The effect of three parameters: *H*, *C*_1_,${\text{ and }}\alpha$ got from the log-stable model with the scaling exponent separately is illustrated in Figure 13 for presenting the intermittency and multifractal properties of GHI. According to the classification, the day (11-June-2016) in Figure 13a) is sunny day; the days (18-August-2016 and 2-January-2017) in Figure 13b,d are sunny in the morning and cloudy in the afternoon; the day (1-April-2017) in Figure 13e is cloudy day, the day (21-October-2016) in Figure 13c is cloudy in the morning and sunny in the afternoon.

### Seasonal variability of daily multifractal pattern

After checking the sub-daily and daily GHI multifractal pattern, it would be interesting to see the GHI variability throughout the year and especially through the seasonal cycle (month by month). This section is to show the multifractal fluctuation of the GHI through the day month by month.

**Figure 14:**
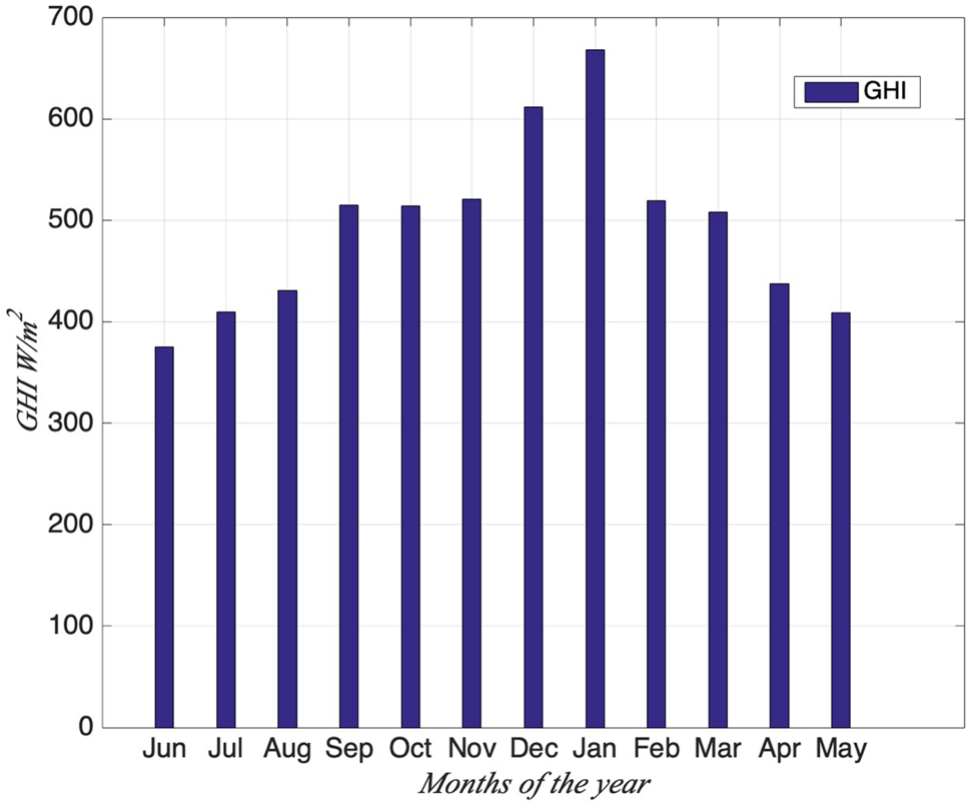
Monthly mean of global solar radiation from June 2016 to May 2017 (dataset sampling on second).

Reunion Island is a tropical island on the south hemisphere and months from November to April are in austral summer (the warm and humid season), May to October are austral winter (the fresher and drier season). Normally the austral winter comes in July and August, and the austral summer reaches its peak in January and February. Based on one-year second data from June 2016 to May 2017 at station Moufia, here the analysis on seasonal variability of daily multifractal pattern is present depending on these dataset (month by month). Figure 14 shows the distribution of monthly GHI from June 2016 to May 2017. The lowest monthly value of global solar radiation appears in June 2016 and the highest in January 2017 in Figure 14.

**Figure 15:**
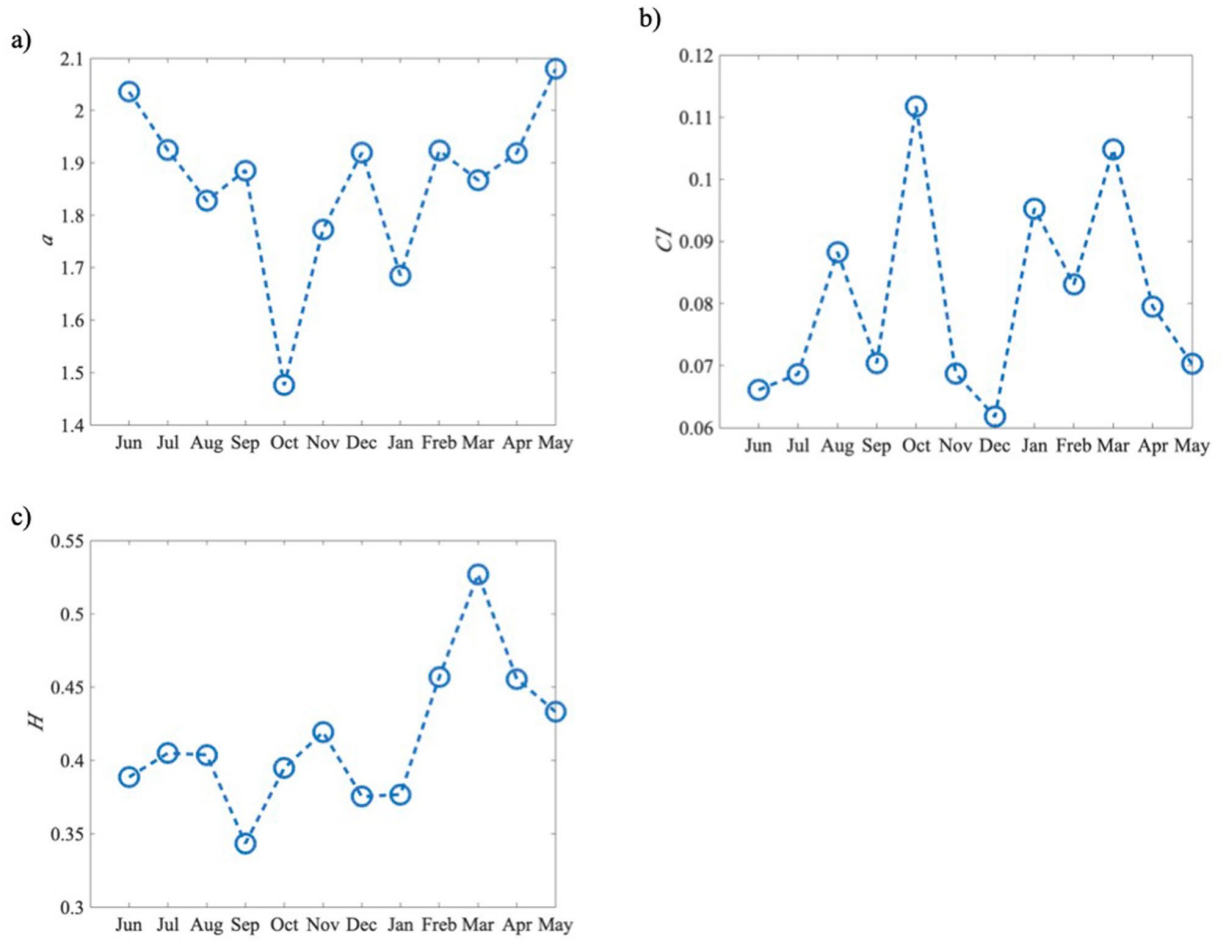
Universal multifractal parameter *H*, *C*_1_,${\text{ and }}\alpha$ in each month from June 2016 to May 2017.

Figure 15 shows the change of the universal multifractal parameter *H*, *C*_1,_${\text{ and }}\alpha$ in each month from June 2016 to May 2017. Firstly, the monthly change for these three parameters indicates the seasonal variability of the multifractal pattern for GHI. The monthly mean of *H* over the one year is lower than 0.5 (anti-persistent), except the month May whose *H* is bigger than 0.5 (persistent). *α ∈ *[1, 2] during July 2016 to April 2017 indicates that GHI of those months are multifractal with Levy generators and unbounded singularities. The monthly *C*_1_ values are quite small in May, June and December which are inter-season months, normally they are more intermittent, but here they are more homogeneous for GHI than other months.

## Conclusion and discussion

The arbitrary order Hilbert spectral analyses which is the combination of the Empirical Mode Decomposition and Hilbert spectral analysis (EMD + HSA) are applied to study the intermittency and multifractality of GHI over Reunion Island in this paper. The scaling exponents $\xi \left( q \right)$ is estimated through the arbitrary order Hilbert spectral analyses and three parameters (*H*, *C*_1_,${ }\alpha$) are taken to study the multifractal process of the GHI. One available second sampling rate of 1-year GHI records (one observation per second) are used to achieve the multifractal analysis.

The GHI during the day time (7–17 h) sampling on second at one station Moufia from June 2016 to May 2017 are applied to obtain the generalized scaling exponent and log-stable model parameters. The multifractal processes could be found in the sub-daily and daily fluctuations. A power law behavior with a spectral exponent *β* = 1.68 close to the Kolmogorov spectrum is detected through Fourier spectrum analysis in this GHI time series, which also indicates that the sub-daily fluctuations of GHI are nonstationary. The scaling exponent *ζ*(*q*) is then estimated by the arbitrary order Hilbert spectral analysis and the multifractal properties is detected. The log-stable model parameters $H,{ }C_{1}$ and $\alpha$ characterize the concavity of the scaling exponent *ζ*(*q*) for analyzing the intermittency of GHI. *H*-the Hurst parameter defines the degree of smoothness or roughness of the field; secondly, *C*_1_ measures the inhomogeneity mean or the mean intermittency characterizing the sparseness of the field and thirdly, and the multifractal Lévy parameter α measures the degree of multifractality. The classification method is applied to the daily GHI for analyzing the distribution of the daily intermittency process and five classes with GHI and *K*_*b*_ are obtained. The multifractal parameters (*H*, *C*_1_,${ }\alpha$) then are classified into these five classes which present the variability of intermittency for each type of weather and thus the range value of (*H*, *C*_1_,${ }\alpha$). The monthly distribution of the multifractal parameters (*H*, *C*_1_,${ }\alpha$) also indicate the seasonal variability of daily GHI.

Calif et al.^18^ studied the intermittency of global solar radiation over one year at Guadeloupean Archipelago. Several multifractal analysis methods, including multifractal detrended fluctuation analysis, wavelet leader, structure functions, arbitrary order Hilbert spectral analysis, have been applied to global solar radiation sequences in their paper. In this paper, just the newer approach—the arbitrary order Hilbert spectral analysis has been used to present the multifractal parameters (*H*, *C*_1_,${ }\alpha$), which give the similar characterization of intermittency of solar radiation sequences. Different to Calif et al.^18^, the classification method is applied to the multifractal parameters (*H*, *C*_1_,${ }\alpha$) according to the clear sky index of global solar radiation, which can present the different weather condition based on the range value of multifractal parameters. Beside of the daily variability analysis as Calif et al.^18^, the sub-daily and seasonal variability analysis of daily multifractal pattern are also discussed in this paper. Thus, the arbitrary order Hilbert spectral analysis combined with the classification method not only provides a way to analyze the intermittency and variability of the solar radiation, but also can serve as a new method to give predictors describing intermittency for the solar radiation forecast.
