# Author Correction: Intermittency study of global solar radiation under a tropical climate: case study on Reunion Island

**DOI:** 10.1038/s41598-022-27363-9

**Published:** 2023-01-09

**Authors:** Qi Li, Miloud Bessafi, Peng Li

**Affiliations:** 1 Guangxi University of Science and Technology, 268 Avenue Donghuan, Chengzhong District, Liuzhou, Guangxi China; 2 ENERGY Lab, Université de La Réunion, rue René Cassin, St Denis, 97715 France

Correction to: *Scientific Reports* 10.1038/s41598-021-91639-9, published online 09 June 2021

Miloud Bessafi was omitted from the author list in the original version of this Article.

The Author Contributions section now reads:

“L.Q., M.B. and L.P. wrote the main manuscript. All authors reviewed the manuscript. M.B. contributed with the resources, funding, acquisition and was the lead supervisor. L.P. as the corresponding author charged the submission and contact process.”

In addition, the Acknowledgements section in the original version of this Article was incomplete.

“We thank the lab LE2P in the University of Reunion for providing surface solar radiation dataset. We thank Professor Miloud Bessafi and Professor Huang Yongxiang for helping on the method of the arbitrary order Hilbert spectral analysis.”

now reads:

“We thank the lab LE2P in the University of Reunion for providing surface solar radiation dataset. We thank Professor Huang Yongxiang for helping on the method of the arbitrary order Hilbert spectral analysis. This work was supported by the Europe and Region Reunion (Tiers: 181727-Dossier N: D2013032473).”

The original Article has been corrected.

